# Content validation of a mental wellness measuring instrument for adolescents living with HIV: a modified delphi study

**DOI:** 10.1186/s40359-023-01350-9

**Published:** 2023-10-17

**Authors:** Zaida Orth, Brian van Wyk

**Affiliations:** School of Public Health, Faculty of Community and Health Sciences, University of the Western Cape Robert Sobukwe Rd, 7535 Bellville, South Africa

**Keywords:** Adolescents living with HIV, Instrument design, Content validity, Delphi Study

## Abstract

A growing body of evidence suggests that improving the mental wellness of adolescents living with HIV (ALHIV) will also result in improved adherence to antiretroviral therapy (ART), as well as improving their general health and wellbeing as they age into adulthood. However, to develop effective strategies and interventions aimed at improving mental wellness, we require age and culturally appropriate instruments to build an evidence base. Currently, there is a lack of mental wellness measures developed for ALHIV, especially in the African context. To address this gap, we developed a measure of mental wellness following modified guidelines set out by DeVellis [1] and Godfred et al. as a guiding framework [2]; (1) Identifying the gap, (2) Set the theoretical foundations and identify domains and (3); Instrument development and initial validation. For the first two steps, we conducted a systematic review, photovoice study and integrative review – which we briefly describe as the findings have been published. Following this we describe the processes to develop the instrument and to establish content validity through a modified Delphi Study. Through this process we were able to refine the instrument which will be subject to further testing.

**Implications and Contribution**: This study aims to add to the body of knowledge on promoting mental health (mental wellness) among adolescents living with HIV in South Africa through developing an appropriate and valid measure of mental wellness for this population. This study reports on the results of a Delphi Study aimed at improving the content validity of the instrument Mental Wellness Measure for Adolescents Living with HIV (MWM-ALHIV).

## Background

The improved access and efficacy of anti-retroviral therapy (ART) and the scale-up of treatment programmes and differentiated services have resulted in various gains made in the fight against HIV [3]. Along with advances in medical treatment, advocacy campaigns such as ‘Undetectable = Untransmissible’ (U + U) are changing the narrative around HIV as a deadly infectious disease, to a chronic condition that can be managed through appropriate treatment and intervention [4]. However, despite these advances, rising challenges related to suboptimal adherence, poor retention in care and developing best practices to help people manage a lifelong condition, ensures that HIV remains a major concern in many regions of the world [5]. This is particularly true of the Sub-Saharan African (SSA) region, as it experiences the highest burden of HIV globally, and is home to majority of the world’s adolescents [5].

Further SSA houses the majority of the global population of adolescents living with HIV [6]. In 2019, approximately 1.7 million adolescents aged 10–19 were living with HIV globally, with a reported 85% of this population living in SSA [6]. ALHIV in SSA are disproportionally affected by HIV which is evidenced in their lower achievement of the 95-95-95 HIV cascade and poorer health outcomes , higher mortality rates, lower adherence rates and treatment failure and retention in care, in comparison to children and adults living with HIV [6]. This suggests that there are ongoing gaps between the treatment cascade and lived experiences of ALHIV.

Improved understanding of the circumstances of influencing treatment outcomes among ALHIV is critical in designing adolescent-friendly health services which can help to improve testing, ART initiation and sustained adherence and retention in care [7]. Young people accessing ART face a variety of complex challenges that are distinct from adults, and which negatively impact adherence to treatment [8, 9]. Studies exploring barriers and facilitators to optimal adherence in adolescents have identified a multitude of factors that span across different levels including the individual, family, peers, health care system and the wider community [7, 10, 11]. Amongst these, [lack of] psychosocial support and higher incidence of mental health problems has been identified as having negative effects on adherence and retention in care among ALHIV [12–15]. Recently, there has been an increased focus on mental health promotion among adolescents as evidence suggests that mental wellness, representing the positive dimension of mental health,is a critical foundation to support overall health and wellbeing across an individual’s life course [16, 17].

Within this context, early intervention is critical given the peak age of onset for majority of mental health disorders occur during adolescents [18]. In South Africa, mental health among adolescents is seemingly in crises as the South African Depression and Anxiety Group (SADAG) reports that prevalence rates of anxiety and depression are high among youth, with suicide identified as one of the leading causes of death in this group [19]. For example, a study conducted in the Western Cape reported high prevalence rates of symptoms of depression (41%), anxiety (16%) and post-traumatic stress disorder (PTSD) (21%), among school learners aged 14–15 years [20]. These numbers are concerning, yet reflect findings from previous studies suggesting that ALHIV are at greater risk of developing mental health problems in comparison to their peers due to the nature of their illness and the challenges associated with managing a stigmatized chronic condition. For example, a study conducted by Kim et al. [21] reported that being bullied for taking HIV medications was associated with depressive symptoms among Malawian adolescents, which in turn was a reported barrier to adherence. Conversely, enhancing mental wellness in ALHIV may lead to better physical health outcomes, including improved treatment adherence and care retention. Research across various populations underscores the connection between mental wellness and improved health. These findings indicate that mental wellness acts as a resilience resource, equipping individuals with the psychological and emotional tools to understand and cope with life’s challenges, protecting against both physical and mental illness. However, despite encouraging findings and increased discourse around mental health promotion, we lack empirical evidence to fully understand the mechanisms of change by which mental wellness pathways contribute to positive health outcomes. This information is crucial not only guide the implementation of tailored treatment programmes which are needed to support the mental health needs and adherence outcomes of ALHIV, but also for securing stakeholder support and ensuring the scalability and long-term sustainability of these programs [22, 23].

Efforts are underway to improve psychosocial support services for ALHIV [23, 35, 37]. However, numerous reviews reveal a scarcity of trials examining interventions that address the mental health needs of ALHIV, especially in LMICs [23, 35, 37]. The lack of a clear distinction between mental wellness and mental illness has led to diverse approaches in implementing mental health promotion and psychosocial interventions, which often involve various components like counseling, family and peer support, and economic empowerment, with different instruments being used to measure similar outcomes [24, 25]. This limited and diverse nature of studies makes it challenging to draw comparisons and determine best practices. [24]. Additionally, although mental health is recognized as extending beyond the absence of illness or symptoms, studies claiming “improved mental health outcomes” frequently focus on symptom reduction instead. For example, Cavazos-Rehg et al. report that family based economic intervention implemented in Uganda as part of the *Suubi Adherence* programme effectively improved mental health among ALHIV participants [14]. However, only 3 domains of mental health were measured: hopelessness, depression and self-concept [14]. This limited and diverse nature of studies makes it challenging to draw comparisons and determine best practices and impedes our ability to make evidenced-based decisions to support optimal treatment outcomes for ALHIV. Therefore, there is a need to invest in research to better understand pathways to improving mental wellness among ALHIV as a way to prevent the development of mental health problems or co-morbid mental disorders and to support optimal adherence across the life course. To this end, we set out to develop and validate a measure of mental wellness specifically for ALHIV.

## Methods

In this section we describe the methods related to the scale construction and initial face and construct validation of the Mental Wellness Measure for Adolescents Living with HIV (MWM-ALHIV). Various scholars have proposed guidelines for scale development which include similar steps. We followed a modified version of guidelines in scale development proposed by DeVellis [1] and Godfred et al. [2],; (1) Identify the gap, (2) Set theoretical foundations and identify the domains (3) Item development and initial validation.Both Godfred and DeVellis offer similar guidelines for instrument development. Godfred’s insights are particularly valuable for practical applications in developing scales for health, behavioral, and social research. On the other hand, DeVellis provides a comprehensive understanding of the theory and application of scale development. We made minor modifications to combine these complementary guidelines to enhance the rigor of our approach.

### Identifying the gap

Before setting out to develop a new instrument, it is necessary to determine if one is needed or if existing instruments may be used [1, 2]. To this end, we conducted a systematic review of mental health instruments used in research with adolescents [25, 26]. A key finding from the systematic review, highlighted that there is a lack of consensus around definitions of mental wellness for adolescent. This finding reflects mental health literature stating that concepts such as ‘mental health’, ‘mental wellbeing’ and ‘mental wellness etc. are often used interchangeably or that ‘mental health’ is often used as a euphemism or synonym for ‘mental illness’ [27, 28]. In measurement, this may represent a problem as mental illness and mental wellness are conceptually different. Additionally, many of the instruments identified in the review were developed in high income countries with adult populations and subsequently validated in other contexts. This is a common strategy in research as developing a new measure is a time-consuming process [29]. However, it may be that the items in these measures are not appropriate for adolescents, much less adolescents living in markedly different contexts [29]. For example, while there may be similarities in adult and adolescent mental health, we need to consider that adolescents are not a homogenous group and their constructions of mental health, or the processes and experiences that influence their mental health are different [25, 26, 29]. Therefore, if we only focus on adapting and validating existing measures, we may miss opportunities to fully understand what mental health means for adolescents.

The systematic review provided us with an overview of mental health (focusing on symptoms of mental illness) and mental wellness instruments used for adolescents living with a physical chronic condition [25] and mental wellness instruments used with adolescents in general [26]. However, the findings from the review indicated that there are few measures developed specifically for adolescents with a chronic condition which may present a gap in the research. We reviewed the included instruments to identify the content domains of mental wellness. The instruments we identified varied from measures of general mental wellness, which included multiple domains such as connectedness, self-esteem, spirituality etc. to instruments measuring specific domains of mental wellness. Additionally, we noted that instruments of Health Related Quality of Life (HRQoL) and Quality of Life (QoL) were used to measure mental wellness in research with adolescents living with a chronic condition.

### Setting the theoretical foundations and identifying the domains

The way in which a construct is defined, determines how it will be measured [27, 28, 30]. Therefore, to develop an effective measure of mental wellness, we needed to have a clear understanding of the theoretical and operational definitions of mental wellness [27, 28, 30]. The findings from the systematic review helped us to build a preliminary framework of mental wellness domains measured in research with adolescents. To test the relevance of these domains, we conducted an exploratory photovoice study with ALHIV. The photovoice study allowed us to explore how ALHIV understand and talk about mental wellness in the South African context and included the perspectives of key healthcare workers working closely with ALHIV. We used the findings from these studies to refine the preliminary framework of mental wellness. To further unpack the mental wellness concepts, we used the framework to guide an integrative review of mental wellness concepts identified in research with ALHIV in the African context. This helped us to define mental wellness as a construct and to delineate the relevant domains to be measured.

Based on the findings from these three studies, we identified six mental wellness concepts: Connectedness, Sense of Coherence (SOC), Self-esteem, Self-acceptance, Hope for the Future, and Spirituality as well as six behaviours facilitating mental wellness: Coping, Resilience, Purpose in Life (goals), Self-efficacy, Adherence Self-efficacy, and Leisure Activities representing 12 proposed domains of mental wellness. From this, we proposed a Salutogenic Model of Mental Wellness (SMoMW), which was modified from Antonovsky’s Salutogenic Model of Health [31]. The concept and theoretical basis of these concepts and how it was used to develop the SMoMW have been previously described in previous publications (refs).

### Instrument development and initial validation

#### Generating an initial pool of items

Once we were satisfied that data saturation had been reached and no new mental wellness concepts were identified following the systematic review, photovoice study and integrative review, we proceeded to the item writing phase. Based on the Salutogenic Model of Mental wellness, the proposed instrument consisted of SOC as an indicator of overall mental wellness measured across 11 sub-domains: Connectedness, Self-esteem, Self-acceptance, Hope for the Future and Spirituality, Coping, Resilience, Purpose in Life (goals), Self-efficacy, Adherence Self-efficacy, and Leisure Activities. To ensure that the content reflected the construct of interest, we noted an operational definition for each sub-domain and then began by generating an initial pool of items by drawing on the instruments already identified from the systematic review. As the items in these instruments were developed with samples of English-speaking adolescents from the Global North, we adapted the phrasing so that it could better reflect the context and comprehension level of ALHIV in South Africa who may speak English as a second additional language. Additionally, we used the findings from the photovoice study to write items for each sub-domain that specifically relate to the context of HIV.

Foxcroft and Roodt [32] suggest including a substantial number of items in the initial phase because many are often discarded during testing. They propose twice the number needed for the final version. DeVellis recommends an even larger initial pool, three to four times the final scale size, to enhance internal consistency [1, 32]. We followed these guidelines and the systematic review, where scales had 5–90 items on average, to establish a baseline of items. For the final measure, at least 5 items per domain are advised [1, 32]. Accordingly, we aimed for 60–110 items in the MWM-ALHIV, starting with 10–15 items per domain, totaling 120–180 items across 11 domains.*Structural decisions*.

We initially proposed a 5-point Likert scale with responses ranging from “Strongly Disagree” to “Somewhat Disagree”, “Disagree”, “Somewhat Agree” and “Strongly Agree” - as this is a widely used instrument to measure attitudes, opinions and beliefs [1, 32]. Furthermore, the Likert scale was deemed as an appropriate choice as the MWM-ALHIV is a self-administered screening instrument which allows for initial evaluation and scoring of mental wellness across the different sub-domains. Additionally, this is considered an appropriate measurement type, as our goal is not to provide a clinical diagnosis of mental health problems, rather a general assessment of positive mental wellness ‘strengths’ which can be reinforced (through intervention programs, workshops etc.) to buffer against the development of mental health problems. Therefore, this screening tool will allow us to categorise participants into groups (e.g. ALHIV with high mental wellness or ALHIV with low mental wellness) with reasonable accuracy and can provide information on how mental wellness is related to maintaining adherence to ART (i.e. are participants with higher mental wellness scores demonstrating adherence?) or may provide an indication of which domains of mental wellness need intervention (i.e. participants may score high in one domain and low in another). Furthermore, categorizing participants as “high mental wellness” and “low mental wellness” may contribute to a better understanding of how mental wellness functions as a continuum which is separate, yet related to mental illness. Hence, higher scores on measures of mental wellness indicate the presence of positive mental health, whereas lower scores signify the absence of positive mental health. Such distinctions can aid in constructing an evidence base that fosters consensus regarding the use of terminology (i.e. mental well-being, positive mental health), addressing ongoing debates about the significance of eudemonic versus hedonic well-being and preventing the confusion of mental illness with mental health. Screening instruments are considered advantageous as they can be easily administered in a timely and cost-efficient manner. Furthermore, the results from screening instruments are immediately available and easy to score and interpret – as such it may be used by a variety of professionals, including health care workers, counsellors, school staff, paraprofessionals and trained volunteers.

#### Target group

The MWM-ALHIV is intended for ALHIV aged 15–19 years old.

#### Development of the stimulus document

The information for the proposed MWM-ALHIV was drafted into a stimulus document to be reviewed by experts in the field (step 3). The stimulus document included 2 sections – the first presented information around the definitions of the domains, aim of the measure, measure type and other technical aspects. The second section provided information around the actual instrument and listed the proposed items under each domain. Participants were prompted to give qualitative feedback on each section by providing a space where they could share additional comments that may not have been addressed in the document and to select items which they thought were the most relevant. Additionally, participants were prompted to provide feedback or suggest any changes to the items.

### Expert review of items

Involving experts in the field and service users in the instrument development process is crucial to maximizing the content validity and establishing the usability of the instrument [29, 33]. Therefore, we invited a group of experts in the fields of HIV, adolescent mental health, and instrument development to participate in a modified Delphi study and provide feedback on the proposed instrument through the stimulus document. Additionally, after the completion of the Delphi Study, we aim to conduct Cognitive Interviews with another group of experts namely, ALHIV aged 14–19 whom the instrument is intended.

#### Delphi study participants and procedures

Through a process of snowball sampling, 20 experts were contacted and invited to take part in the Delphi Study. Each participant was contacted via email and received an information sheet and consent form. Those who expressed their willingness to participate were requested to return the signed consent form. Subsequently, they received the stimulus document along with a demographic questionnaire, which was designed to collect descriptive information about the participants. Participants were asked to provide feedback within 2 weeks of receiving the document. Of the 20 invitations we sent out, 15 consented to participate in the study and 11 returned the stimulus document with their feedback in the 1st round. The responses from the 11 participants were captured in an excel sheet and analyzed to adjust the stimulus document for the second round. In the second round, the 11 participants received the updated stimulus document to provide feedback. After two weeks, 5 participants responded with their feedback for the 2nd round. We sent a follow up email to those who did not respond – after which 3 more participants responded and three were counted as non-response. Therefore, a total of 8 participants responded to the second round.

#### Data analysis

For the 1st round of the Delphi Study, consensus was defined as ≥ 70% (N = 8) and in the second round it was defined as ≥ 50% (N = 4) which is considered appropriate [34].

### Ethics

This project, which forms part of the first-author’s doctoral research project, follows the ethical principles set in the Declaration of Helsinki (1964) and received ethical clearance from the University of the Western Cape Biomedical Research Ethics committee (BM19/09/18). All participants were contacted via email and the study was explained in detail, including the nature of participation. Those who indicated interest received an information sheet and consent forms. All participants provided written informed consent by returning the signed form before proceeding to participate in the study.

## Results

In the first round of the Delphi study 11 (73%) of invitees completed the survey. Of the 11 participants, 8 (72%) completed the second round of the survey. The characteristics of the participants are presented in Table 1. The majority of participants possess over a decade of experience in their respective fields. Notably, many participants hold current positions in academia, such as researchers or lecturers. However, that is not indicative of the extent of their experience. As is the nature of the field, participants also indicated that their work is interdisciplinary and extends beyond academia to encompass various professional engagements.

**Table 1:** Demographic characteristics of delphi participants

Demographics	Round 1 (n = 11)	Round 2 (n = 8)
Qualification		
PhD	8	6
MD	1	
Psy.D. Clin Psy	2	2
Years of experience		
1–10	3	1
> 10	8	7
Areas of expertise		
HIV	5	4
Adolescent health	4	2
Adolescent mental health	5	3
Epidemiology & statistics	1	1
Instrument design	7	5
Sectors		
Academic/Higher education	10	6
Medical center/health facility	4	4
Private sector	2	2
NGO	3	2
Current profession		
Clinician	1	1
Researcher	7	5
Lecturer/Prof	4	3
Mental health specialist/Clinical Psychologist	3	3

In Table 2, we provide a summary of the agreement on the 1st section of the stimulus document, while Table 3 provides an overview on how the domain definitions were changed after integrating the feedback As shown in the tables below, although there was a high level of agreement, most participants suggested modifications.to improve the clarity of the domain definitions or other technical aspects of the instrument. These suggestions were used to edit the stimulus document accordingly resulting in even greater consensus during the second round. In the first round, participants indicated that a Likert-scale was appropriate, however there was some disagreement regarding the number of responses on the scale. Based on the suggestions, we proposed a 4-point Likers-scale in the second round which all the participants agreed with. The second round indicates overall improvement as almost all participants endorsed the changes – with the exception of two minor comments for the definitions of self-efficacy and coping. After the second round, consensus for all stimulus prompts was reached, therefore it was determined that a third round would not be needed.

**Table 2:** Summary of consensus among participants

Statements relating to the purpose and aim of the instrument	Round 1 (N = 11)		Round 2 (N = 8)	
	Yes	Suggest changes	Yes	Suggest changes
Is the definition of self-acceptance clearly stated?	7 (64%)	4 (36%)	8 (100%)	0
Is the definition of self-esteem clearly stated?	7 (64%)	4 (36%)	8 (100%)	0
Is the definition of self-efficacy clearly stated?	8 (73%)	3 (27%)	7 (88%)	1 (12%)
Is the definition of adherence self-efficacy clearly stated?	9 (82%)	2 (18%)	8 (100%)	0
Is the definition of resilience clearly stated?	9 (82%)	2 (18%)	8 (100%)	0
Is the definition of coping clearly stated?	8 (73%)	3 (27%)	7 (88%)	1 (12%)
Is the definition of connectedness clearly stated?	9 (82%)	2 (18%)	8 (100%)	0
Is the definition of leisure activities clearly stated?	7 (64%)	4 (36%)	8 (100%)	0
Is the definition of spirituality clearly stated?	8 (73%)	3 (27%)	8 (100%)	0
Is the definition of hope clearly stated?	9 (82%)	2	8 (100%)	0
Is the definition of purpose in life clearly stated?	8 (73%)	3 (27%)	8 (100%)	0
Is the aim of the measure clearly stated?	9 (82%)	2 (18%)	8 (100%)	0
Is the purpose of the instrument clear?	10 (91%)	1 (9%)	8 (100%)	0
Is the type of instrument appropriate?	9 (82%)	2 (18%)	8 (100%)	0
Is the type of item (5-point Likert type) an appropriate choice for the screening instrument?	4 (36%)	7 (64%)	8 (100%)	0
Is the user group clearly defined?	7 (64%)	4 (36%)	8 (100%)	0
Is the target population clearly stated?	10 (91%)	1 (9%)	8 (100%)	0

In Table 4, we provide an overview of changes to the number of items after each round of the Delphi Study. In both rounds, items which did not reach consensus were discarded. As shown in the table, some of the domains had less items in the second round while some domains had more items. This was due to the qualitative feedback and suggestions given by the participants during the first round which we included in the second round. After the second round, all items that received ≥ 50% consensus were retained as the final version of the instrument.

**Table 3:** Conceptual and operation definition changes of each domain following feedback from participants

Domain	Domain Definition Round 1	Domain Definition Round 2	Final Definition
**Self-acceptance**	-A positive attitude toward yourself; acknowledge and accept multiple aspects of yourself including both good and bad qualities; and feel positive about your past life. -based on internal validation - ALHIV accept themselves and overcome internalised stigma related to HIV	-Acknowledging and embracing every aspect of yourself including both good and bad qualities as you accept that this forms part of who you are. Therefore, it involves developing a realistic sense of self (understanding) – a subjective awareness of your strengths, weaknesses and circumstances - To be self-accepting implies accepting yourself unconditionally in the present despite past behaviours or circumstances. It involves accepting parts of ourselves that we may not necessarily like, without offering justification for any of them (e.g., ALHIV do not need to feel the need to justify their status, as indicated in our study on mental wellness discourses, ALHIV needed to overcome internalised stigma around HIV to accept themselves as ‘beautiful’ and ‘normal’ regardless of their status) (Orth & van Wyk, 2022), as well as acknowledging the worthiness that is inherent in each one of us despite our flaws and shortcomings. - Research shows that self-acceptance and self-esteem are conceptually different, yet closely related constructs. While both self-acceptance and self-esteem seem to show positive correlation with mental health outcomes, studies suggest that self-esteem is more closely related to affect, while self-acceptance relates to general psychological wellbeing (Popov, 2018). A high level of self-esteem is a prerequisite for self-acceptance.	No changes
**Self-esteem**	A person’s overall subjective sense of personal worth or value -Self-esteem is based on external feedback and validation, therefore may fluctuate -ALHIV with high self-esteem talk about themselves in a positive light, highlight their best qualities	-Self-esteem is based on how we see ourselves in relation to our own expectations (internal assessments) and with others (external feedback and validation). Self-esteem can be influenced by many factors within our environment, especially during adolescence, such as socializing with others, school performance, self-efficacy etc. -Unlike self-acceptance, self-esteem can be divided into high or low self-esteem. -ALHIV with high self-esteem talk about themselves in a positive light, highlight their best qualities, feel confident and good about themselves, make choices that reflect a positive self-view	No changes
**Self-efficacy**	-A person’s particular set of beliefs that determine how well one can execute a plan of action in prospective situations. Self-efficacy is a person’s belief in their ability to succeed in a particular situation.	-Self-efficacy is a person’s belief in their ability to succeed in a particular situation. It includes the belief in one’s ability to overcome and effectively negotiate life challenges and to complete goals and aspirations.	
**Adherence self-efficacy**	-Belief in one’s ability to successfully adhere to treatment plans -ALHIV can successfully manage their treatment by taking it correctly and on time every day	-Belief in one’s ability to successfully adhere to treatment plans which may include ALHIV successfully managing their treatment by taking medication correctly and on time every day, maintaining appointments, collecting medication and observing other requirements outlined by their healthcare providers. Additionally, ALHIV can maintain their treatment plan even if they experience a change in circumstances or new stressors.	No changes
**Resilience**	-The ability to mentally withstand or adapt to uncertainty, challenges, and adversity. -Includes having access to knowledge and resources to withstand or adapt to stressors.	-The ability to mentally withstand or adapt to uncertainty, challenges, and adversity. -The ability, determination, and strength to overcome stressful life situations - The ability to thrive despite obstacles or disadvantageous circumstances -Includes navigating access to knowledge and resources to withstand or adapt to stressors.	No changes
**Coping**	-Coping refers to cognitive and behavioural efforts to manage (master, reduce, or tolerate) a troubled person-environment relationship. -ALHIV may engage in a range of coping mechanisms to deal with daily life stressors as well as those associated with managing a lifelong condition -This may include engaging in activities they enjoy, connecting with others, engaging in meaningful activities.	-Coping refers to positive cognitive and behavioural efforts to manage (master, reduce, or tolerate) daily life stressors in healthy ways. -ALHIV may engage in a range of coping mechanisms to deal with daily life stressors as well as those associated with managing a lifelong condition -This may include engaging in activities they enjoy, connecting with others, engaging in meaningful activities.	Wording change: -This may include engaging in enjoyable or meaningful activities and connecting with others to positively manage, reduce or address stressors.
**Connectedness**	Sense that one has satisfying relationships with others, believing that one is cared for, loved, esteemed, and valued, and providing friendship or support to others -Feelings of belonging -Feeling taken care of, and wanting to take care of others (for ALHIV, this can come in the form of having family remind them to take medication, or reminding their family members to take medication in the case others in the household are HIV+) -ALHIV may experience support from clinic staff -Spending time with friends -Engaging in community activities	-Sense that one has satisfying relationships with others, believing that one is cared for, loved, esteemed, and valued, and providing friendship or support to others -Feelings of belonging and acceptance in different environments and contexts (i.e. family, school, community) -Feeling taken care of, and wanting to reciprocate by taking care of others which also fostered a sense of purpose and meaning (in our study, participants expressed that they felt taken care of when family members reminded them to take medication, or reminding their family members to take medication in the case others in the household are HIV+, wanting to do well in life so that they can support their loved ones) -ALHIV may experience support from family, clinic staff, spending time with friends, engaging in community activities	No changes
**Leisure activities**	Engaging and participating in activities that bring enjoyment. -Like their peers, ALHIV engage in diverse interest and hobbies such as listening to music, sports, reading, dancing etc. these activities help them to cope, connect with others and find enjoyment in life. Additionally, engaging in such activities is indicative of a sense of physical wellbeing.	-Engaging and participating in healthy activities that bring enjoyment and relaxation (de-stress) -Like their peers, ALHIV engage in a range diverse interest and hobbies based on their personal interests, for example - listening to music, sports, reading, dancing etc. these activities help them to cope, connect with others and find enjoyment in life. Additionally, engaging in such activities is indicative of a sense of physical wellbeing.	No changes
**Spirituality**	-Psychological process of bringing one’s attention to the internal and external experiences occurring in the present moment; concern for or sensitivity to things of the spirit or soul. -ALHIV may attend religious ceremonies and activities -Belief in a higher power -Connect with cultural heritage -For ALHIV, spirituality can help them cope with life stressors by finding meaning in situations	-Spirituality is a broad concept comprising of several key attributes, meaning and purpose, transcendence, connectedness, relationships and religiosity (Puchalski et al. 2014). Can include a experiencing a relationship or connection with a higher being or power outside oneself or the psychological process of bringing one’s attention to the internal and external experiences occurring in the present moment, concern for or sensitivity to things of the spirit or soul. Spirituality confers inner strengths, comfort, peace and wellness. Experiencing a sense of inner peace, contentment and happiness with oneself and others. -ALHIV may attend religious ceremonies and activities, express a belief in a higher power, connection with cultural/spiritual heritage and traditions. For ALHIV, spirituality can help them cope with life stressors by finding meaning in situations	No changes
**Hope**	-Emotion characterized by positive feelings about the immediate or long-term future. -ALHIV show a positive attitude toward life and their future -Hopes and dreams for the future may motivate ALHIV to stay on treatment -Some hope to finish school, get a good job, get married, start a family or even a cure for HIV. -Like many adolescents, ALHIV may be idealistic about their future dreams (i.e., wants to be a singer or soccer star etc.).	-Process of thinking about one’s goals, along with the motivation to move towards those goals (agency) and ways to achieve those goals (pathways) Emotion characterized by positive feelings about the immediate or long-term future. Optimistic view of the future and life that will lead to positive outcomes. - For ALHIV it contributes to a “process of meaning-making which enables people to psychologically cope with the stressful life event of HIV infection and helps them to maintain a psychological balance” (Plattner and Meiring 2006: 244). -Indicative in ALHIV showing a positive attitude toward life and their future -Hopes and dreams for the future may motivate ALHIV to stay on treatment. Some hope to finish school, get a good job, get married, start a family or even a cure for HIV. Like many adolescents, ALHIV may be idealistic about their future dreams (i.e., wants to be a singer or soccer star etc.).	No changes
**Purpose in Life**	-You have goals in life and a sense of directedness; feel there is meaning to your present and past life; hold beliefs that give life purpose; and have aims and objectives for living. -More than hope (less idealistic, more realistic), purpose in life represents a sense of purpose in their lives like caring for their younger siblings or family members -Sense of responsibility -Identified goals and understands the steps needed to reach them	- Adolescence is a formative period for cultivating a sense of purpose in life. -You have goals in life and/or a sense of directedness; feel there is meaning to your present, past and future life. Purpose is a part of one’s personal search for meaning, but it also has an external component, the desire to make a difference in the world, to contribute to matters larger than the self. More than hope (less idealistic, more realistic), purpose in life represents a sense of meaning in their lives like caring for their younger siblings or family members, developing goals to achieve future success	No changes

**Table 4:** *Summary showing the number of item changes after each round*

Domains	Number of items in each domain		
	Round 1 (N = 11)	Round 2 (N = 8)	Final list of items
**Self-acceptance**	23	17	10
**Self-esteem**	11	14	9
**Self-efficacy**	10	10	9
**Adherence self-efficacy**	12	15	13
**Resilience**	26	20	10
**Coping**	33	25	10
**Connectedness**	41	31	10
**Leisure activities**	16	10	10
**Spirituality**	13	9	7
**Hope**	41	28	14
**Purpose in life**	13	13	11
	*consensus ≥ 70%	*consensus ≥ 50%	

In Table 5, we present the final instrument and items under each domain. This version of the instrument will be used to conduct Cognitive Interviews with ALHIV receiving treatment at a public healthcare facility in the Cape Town Metropole to ensure that the participants understand the questioning and phrases as intended.

**Table 5:** *Final Instrument*

Domains	Final Items
**Self-acceptance**	When I fail at something important to me, I remind myself that it is part of being human
	I’m kind to myself.
	Even when I am bad at something, I still love myself.
	When something upsets me, it does not change how I feel about myself
	I am a valuable person, even if there are parts of myself that I do not like.
	I am happy with the way I am
	I feel I am a valuable person even when other people don’t like me.
	I am comfortable with who I am as a person
	Living with HIV does not define who I am
	Living with HIV is part of who I am, and I am okay with that
**Self-esteem**	I feel good about myself.
	There are many things that I like about myself.
	I am proud of myself
	I can do things as well as other people my age.
	I do not have much to be proud of.
	I feel that I’m just as worthy as other people my age
	I wish I could like myself.
	I take a positive attitude toward myself.
	When I think about living with HIV, I feel bad about myself
**Self-efficacy**	I can do most things if I try
	There are many things that I do well
	I know my strengths
	Living with HIV has not affected my ability to do what I want in life
	I feel strong
	I have many talents
	There are many things I cannot do because I am living with HIV
	I don’t try new things because I am scared, I will fail
	I doubt myself
**Adherence self-efficacy**	Taking my treatment is part of my daily routine
	I find ways to take my treatment every day, even when I am around people who don’t know that I am living with HIV
	I take my treatment every day even when there are changes in my daily routine (example travelling)
	I take my treatment every day even when I don’t feel well.
	I take my treatment every day even when my eating habits have changed.
	I take my treatment every day even when it gets in the way of my daily activities
	I take my treatment every day even when I am having a bad day
	I take my treatment every day even when I have problems going to the clinic.
	I take my treatment every day even when people close to me tell me not to
	I take my treatment every day even when I feel like people will judge me
	I am confident that I can manage my treatment
	I try to attend all my clinic appointments even when I have problems going to the clinic
	I do other things to try and stay healthy (e.g., eat healthy foods, drink water, exercise)
**Resilience**	I can work out my problems without hurting myself or others (for example by using drugs and/or being violent).
	I know where to go in my community to get help
	I do things at school that make a positive difference (i.e., make things better)
	I do things at home that make a positive difference (i.e., make things better)
	I know where to go for help when I have problems
	In general, I feel I am in control of my life
	For me, life is about learning, changing, and growing despite my circumstances
	I am confident that I can overcome the challenges in life
	Living with HIV does not stop me from accomplishing what I want in life
	When life is challenging, I give up
**Coping**	I try to work out problems by talking about them
	I think it is important to have new experiences that challenge how you think about yourself and the world
	I am good at managing my responsibilities.
	When I have problems, I think about different solutions to solve the problem
	When I have problems, I take action to solve it
	I try to avoid difficult situations as much as possible
	When I think of living with HIV, I remind myself that there are others who are like me
	I ask others for help when I need it
	When I feel sad or when something is bothering me, I try to think of something else
	When I feel sad, or when something is bothering me, I do activities I enjoy (e.g., playing games, listening to music, reading etc.).
**Connectedness**	I talk to my family/caregiver(s) about how I feel
	My family has fun together
	I feel close to my sibling(s) or other family member(s) (leave blank if you have none)
	I feel comfortable around my family
	Someone in my family accepts me for who I am
	I have friends I’m really close to and trust
	I feel like I belong in my community
	My family stands by me during difficult times
	I feel supported by the clinic/hospital staff
	I feel all alone in the world
**Leisure activities**	I am able to do the activities I enjoy most
	I have enough time for myself
	I am able to do the things I want to do in my free time
	My daily life has been filled with things that interest me
	My daily activities often seem boring and unimportant to me.
	When I do an activity, I enjoy it so much that I lose track of time.
	I get so involved in activities I enjoy that I forget about everything else.
	It is important to me that I feel satisfied by the activities that I take part in.
	Living with HIV has not stopped me from doing the things I like
	I have no energy to do the things I like
**Spirituality**	When I feel lost, I look to my religious leader/elders/culture for help.
	My faith or spiritual beliefs are a source of strength for me
	I participate in organized religious activities
	I feel a sense of peace within myself
	I find comfort in my faith or spiritual beliefs
	I find strength in my faith or spiritual beliefs
	Living with HIV has strengthened my faith or spiritual beliefs
**Hope**	I will accomplish what I want to do in my life
	I will find good work
	I will find work I enjoy
	When I grow up, I will have a good family life
	When I grow up, I will have the life I want
	I will have good health
	I will have a long life
	I will be able to provide for myself
	I will be able to provide for my family
	I am optimistic about my future.
	I think that good things are going to happen to me.
	I believe that things will work out
	I am living with HIV, and I have a bright future
	Living with HIV will not stop me from achieving my goals
**Purpose in life**	Getting an education is important to me
	I think it is important to help out in my community
	I have goals and plans for the future
	I feel a sense of purpose in my life
	I have a reason for living
	I enjoy making plans for the future and working to make them a come true
	I have a sense of direction in life.
	I can say that I have found what I want to do in life
	I am learning more about myself and who I want to be
	I struggle to think of what I want in life
	I have no goals or aims

## Discussion

There is a growing recognition of the importance of mental wellness and mental health promotion to improve overall health and wellbeing [28, 35]. For example, the importance of this issue is evidenced in the Sustainable Development Goal (SDG) agenda, indicating that the mental health (wellness) promotion is essential to reduce the rate of premature deaths [36]. This is especially salient among ALHIV as evidence suggests that interventions aimed at improving their mental wellness are associated with improved rates of adherence to ART and retention in care [23, 37, 38]. Measurement is an essential part of the process – if we cannot effectively measure a social construct such as mental wellness, we will not be able to develop appropriate responses or strategies to improve and strengthen mental health promotion.

According to Krause [29] and DeVillis [1] researchers tend to focus on the technical aspects of instrument development or how data is analyzed while overlooking the importance of being well grounded in the theories or conceptualization of the phenomena being measured. Through using multiple methods, such as the reviews and photovoice studies, we were able to conceptualise and operationally defining mental wellness among ALHIV. As noted in the literature, developing a new measure following this strategy is a time-consuming process – however, it allowed us to identify which domains of mental wellness are most relevant for ALHIV in the South African context and to develop a tentative theoretical model of mental wellness to guide the development of the instrument. Through the modified Delphi Study, we were able to engage with a range of academic and clinical experts to provide valuable feedback on the proposed instrument which helped us to adjust the type of scale to be more user-friendly to adolescents, and to refine the list of items which would be most appropriate for ALHIV in the South African context. Therefore, the Delphi study helped to establish the initial content validity of the mental wellness measure for ALHIV. This information will be used to prepare us to engage with the next round of experts – ALHIV for who this instrument is intended.

The MWM-ALHIV includes both eudemonic and hedonic dimensions of mental wellness. Previous scholars have argued that there is a need for empirical studies that go beyond life satisfaction and well-being in adolescents, to include both eudemonic and hedonic dimensions of mental wellness, explore the relationship between these dimensions and how this can be integrated into a meaningful framework to address mental wellness in a holistic manner [39–41]. Therefore, we argue that the key strength of this measure lies in its use of multiple methods to conceptualize and define mental wellness. Once its psychometric properties are established, it can serve as a valuable tool for conducting empirical research, contributing to the evidence base on mental wellness, including both eudemonic and hedonic dimensions, among ALHIV in the South African context.

## Conclusion

Interventions and mental health services aimed at improving mental wellness among ALHIV requires are more effective when they are tailored to the unique mental wellness needs of this population. However, appropriate instruments are needed to provide an evidence base to guide implementation as well as monitoring and evaluation of services and interventions for ALHIV. Our aim in developing the MWM-ALHIV is to bridge the gap between evidence and practice. Simultaneously, we are documenting the process to increase transparency, enabling others to understand the underlying design rationale and facilitating potential adaptations for diverse contexts. In this paper, we emphasize the significance of involving diverse experts, whose input played a vital role in enhancing the initial content validity. This process laid the foundation for subsequent stages of instrument development, making it more streamlined and effective. The Delphi study allowed participants to provide candid feedback, highlighting vital insights and potential biases in the initial measure draft. In the social sciences, achieving complete bias-free measures can be challenging due to the nuanced nature of the concepts we aim to define and assess. Thus, involving a diverse panel of experts for iterative feedback and consensus-building proved to be a crucial strategy to proactively address any issues within the MWM-ALHIV at an early stage.

The findings from the Delphi study provide initial support that the MWM-ALHIV effectively encompasses all relevant domains to capture mental wellness among ALHIV and that the proposed items presented are adequately in tapping into each of those domains. By engaging with a range of academic and clinical experts, we were able to address problems early on to improve the measure’s user-friendliness and appropriateness within the South African context. In the following stages of this research, we will advance the content and face validity of the measure by engaging with a sample of ALHIV using cognitive interview techniques. Once the foundations of sound content and validity have been established, we aim to pilot the MWM-ALHIV to establish further psychometric properties.

## Data Availability

The datasets generated and/or analysed during the current study are not publicly available due to the ongoing nature of the study but are available from the corresponding author on reasonable request.
